# AGE-ASSOCIATED INCREASE IN KYNURENINE SUPPRESSES AUTOPHAGY AND PROMOTES APOPTOSIS IN MESENCHYMAL STEM CELLS

**DOI:** 10.1093/geroni/igz038.401

**Published:** 2019-11-08

**Authors:** Dmitry Kondrikov, Ahmed Elmansi, Robert T Bragg, Tanner Mobley, Meghan mcGee-Lawrence, Mark Hamrick, Carlos Isales, William D Hill

**Affiliations:** 1 Medical University of South Carolina, Charleston, South Carolina, United States; 2 Augusta University, Augusta, Georgia, United States

## Abstract

The age-related increase of the tryptophan metabolite, kynurenine (KYN), has been associated with osteoporosis progression. Increased activity of by Indoleamine-(2,3)-dioxygenase(IDO), are responsible for the elevation of KYN levels in bone tissue. IDO activity is elevated with age and could be a promising therapeutic target forosteopenia and osteoporosis. Previously, our group has shown that the serum level of KYN is elevated with age and correlates with bone loss in vivo. Kynurenine suppress the expression and activity of chemokine CXCL12 essential for osteogenesis, bone marrow stem cells homing. Bone Marrow Stem Cells (BMSC) cultured in 1% FBS were treated with CXCL12 (100ng/ml) in the presence of saline control or the autophagic flux-inhibition agent chloroquine (CQ). CXCL12 treatment increased autophagy by upregulating the degree of LC3B-II by 20%. CXCL12 treatment also significantly increased co-localization of LC3B and LAMP-2 in serum starved cells. In the present study, we tested the theory that kynurenine plays an opposite role to CXCL12 by suppressing the autophagy cell survival pathway and by inducing apoptosis. Treatment of nutrient-deprived murine BMSCs with 10 or 100 µM of KYN suppresses autophagy in a dose dependent fashion while increasing cellular apoptosis. Treatment of BMSCs with KYN downregulated autophagic flux in BMSC preventing CQ-induced CL3B/LAMP-2 colocalization. KYN treatment prevented conversion of LC3B-I to LC3B-II in CQ-treated cells by 30 percent. At the same time, KYN treatment induces apoptosis, by increasing TUNEL-positive cells number by more than 50 percent. Additionally, KYN treatment significantly increased the levels of cleaved isoforms of PARP and caspase-3.

